# HER2 Mediates PSMA/mGluR1-Driven Resistance to the DS-7423 Dual PI3K/mTOR Inhibitor in PTEN Wild-type Prostate Cancer Models

**DOI:** 10.1158/1535-7163.MCT-21-0320

**Published:** 2022-01-27

**Authors:** Valentí Gómez, Myria Galazi, Gregory Weitsman, James Monypenny, Fahad Al-Salemee, Paul R. Barber, Kenrick Ng, Richard Beatson, Bálint Szokol, László Orfi, Greg Mullen, Bart Vanhaesebroeck, Simon Chowdhury, Hing Y. Leung, Tony Ng

**Affiliations:** 1 UCL Cancer Institute, University College London, London, United Kingdom.; 2 School of Cancer and Pharmaceutical Sciences, King's College London, London, United Kingdom.; 3 School of Biomedical Engineering & Imaging Sciences, King's College London, London, United Kingdom.; 4 Vichem Chemie Ltd., Veszprém, Hungary.; 5 Department of Pharmaceutical Chemistry, Semmelweis University, Budapest, Hungary.; 6 Guy's, King's, and St. Thomas' Hospitals, and Sarah Cannon Research Institute, London, United Kingdom.; 7 Cancer Research United Kingdom Beatson Institute, Bearsden, Glasgow, United Kingdom.; 8 Institute of Cancer Sciences, College of Medical, Veterinary and Life Sciences, University of Glasgow, Bearsden, Glasgow, United Kingdom.

## Abstract

Prostate cancer remains a major cause of male mortality. Genetic alteration of the PI3K/AKT/mTOR pathway is one of the key events in tumor development and progression in prostate cancer, with inactivation of the PTEN tumor suppressor being very common in this cancer type. Extensive evaluation has been performed on the therapeutic potential of PI3K/AKT/mTOR inhibitors and the resistance mechanisms arising in patients with PTEN-mutant background. However, in patients with a PTEN wild-type phenotype, PI3K/AKT/mTOR inhibitors have not demonstrated efficacy, and this remains an area of clinical unmet need. In this study, we have investigated the response of PTEN wild-type prostate cancer cell lines to the dual PI3K/mTOR inhibitor DS-7423 alone or in combination with HER2 inhibitors or mGluR1 inhibitors. Upon treatment with the dual PI3K/mTOR inhibitor DS-7423, PTEN wild-type prostate cancer CWR22/22RV1 cells upregulate expression of the proteins PSMA, mGluR1, and the tyrosine kinase receptor HER2, while PTEN-mutant LNCaP cells upregulate androgen receptor and HER3. PSMA, mGluR1, and HER2 exert control over one another in a positive feedback loop that allows cells to overcome treatment with DS-7423. Concomitant targeting of PI3K/mTOR with either HER2 or mGluR1 inhibitors results in decreased cell survival and tumor growth in xenograft studies. Our results suggest a novel therapeutic possibility for patients with PTEN wild-type PI3K/AKT-mutant prostate cancer based in the combination of PI3K/mTOR blockade with HER2 or mGluR1 inhibitors.

## Introduction

Prostate cancer is the second most commonly occurring cancer in men with about 1.3 million new cases diagnosed worldwide in 2018 (1). Most cases present at an early stage and can be treated with curative intent. However, it has been estimated that up to 30% of patients with prostate cancer will develop metastases at some point in their disease course (2). This is mainly due to an alternative activation of androgen receptor (AR) by multiple signaling pathways that allows bypassing the hormone-dependent route (3). Consequently, novel antiandrogen strategies such as enzalutamide and abiraterone acetate have been developed in recent years (4).

Prostate-specific membrane antigen (PSMA, also known as folate hydrolase I or glutamate carboxypeptidase II) is a type-2 transmembrane protein. Its expression is transcriptionally repressed by AR activity. Therefore, as malignant transformation occurs, antiandrogen therapy is administered and AR signaling diminished, prostate cancer cells frequently show increased plasma membrane PSMA expression (5). In these conditions, PSMA exerts both ligand-induced endocytic transport and glutamate-releasing enzymatic activities (6), the latter being shown to correlate with tumor progression (7) upon activation of mechanisms dependent on the seven-domain transmembrane protein G–coupled metabotropic glutamate receptors (mGluR; refs. 8, 9). Despite its specific functions remaining unclear, PSMA has been the object of extensive research as a diagnostic and therapeutic target (10). In addition, the mGluR1 allosteric inhibitor Riluzole promotes AR degradation via an endoplasmic reticulum stress response (11), adding another level of cross-talk between AR and PSMA.

One of the main signaling routes that control AR biology is the PI3K—AKT/protein kinase B—mTOR (PI3K-AKT-mTOR) pathway, with a key role for the PI3Kβ isoform (12). This signaling axis controls multiple cellular processes including metabolism, motility, proliferation, growth, and survival and is counteracted by the PTEN tumor suppressor. Aberrations in PI3K pathway signaling have been identified in approximately 40% of early prostate cancer and in 70%–100% of advanced disease. PTEN loss (either through gene loss or other mechanisms such as inactivating point mutations) results in constitutive PI3K pathway activation and accounts for 30% of primary and 60% of castration-resistant prostate cancers (CRPC; refs. 3, 13–15). Consequently, there has been extensive interest in using PI3K-AKT-mTOR pathway inhibitors, also in combination with radiotherapy (16) or androgen-deprivation therapies (17). Of particular interest are compounds that target more than one PI3K pathway effector in a simultaneous fashion such as dual PI3K-mTOR inhibitors (18, 19).

Recently, the IPATential150 phase III trial demonstrated that ipatasertib, an AKT inhibitor, prolonged survival in patients with PTEN-loss tumors (20). This fulfilled an area of clinical unmet need for patients with PTEN-mutant metastatic CRPC. However, up to 60% of patients in this disease setting do not carry a PTEN mutation, and failed to benefit from the strategy of AKT inhibition in an earlier phase II trial (15). Interestingly, there is a category of patients displaying clonal activating mutations in *PIK3CA/AKT1* with low-level *AR* copy gain and mutually exclusive mutational status between *AKT1* and *PTEN* (21). Therefore, there is a potential scenario for patients that do not present PTEN loss of function, to benefit from PI3K/AKT/mTOR inhibition although the molecular events leading to resistance in this context remain largely unknown.

The current study shows that, in response to the dual PI3K/mTOR inhibitor DS-7423 (22), PTEN-wt prostate cancer cells activate oncogenic pathways dependent on HER2 and PSMA-dependent mGluR1. We found that simultaneous blockade of mGluR1 and PI3K/mTOR pathways resulted in decreased cancer cell survival and tumor growth. This suggests that the combined inhibition of mGluR1 alongside PI3K/mTOR could potentially be of benefit for patients with PTEN wild-type prostate cancer.

## Materials and Methods

### *In vivo* xenograft studies

CWR22 (5 × 10^6^ cells) mixed (1:1) with Matrigel Matrix (Corning) were injected subcutaneously into 6-week-old BALB/c nu/nu male mice (Charles River Laboratories). Two weeks after injection, when tumors reached an appropriate volume of approximately 100 mm^3^, mice were randomized into control and treatment arms. Mice were treated orally (1 dose/day, 10 doses with a 2-day break after dose 5) with the indicated drug combination: DS-7423 (Daichi Sankyo; 3 mg/kg), lapatinib (GW-57201, Biovision; 70 mg/kg), riluzole (A10795-200, Generon, Slough, United Kingdom; 10 mg/kg). Tumor dimensions were measured using caliper and the tumor volume was calculated following the formula *V* = (x*y*z)*π/6. All animals were maintained under specific pathogen-free conditions and handled in accordance with the Institutional Committees on Animal Welfare of the UK Home Office (The Home Office Animals Scientific Procedures Act, 1986). All animal experiments were approved by the Animal Welfare and Ethical Review Body at University College London (UCL, London, United Kingdom) and carried out under license from the UK Home Office.

### PET scanning

PET imaging was performed as described previously (23). Briefly, mice were administered radiolabeled 68Ga-THP-PSMA and imaged under isoflurane anesthesia with a BioScan nanoPET-CT PLUS (Mediso). Radiochemical purity was checked by ITLC (>95%). Mice were CT imaged before radiotracer administration and dynamic PET data were collected for the first hour post-injection. Mice were then euthanized, and organs harvested, weighed, and gamma counted. The dynamic PET data were reconstructed using Nucline software (v 2.00). Image processing and analysis were performed using Vivoquant software (v3.5).

### Cell culture, chemicals, treatments, and transfections

The LNCaP, DU145, PC3, and CWR22 cell lines were maintained in RPMI medium supplemented with 10% FBS. 22Rv1 cells were maintained in phenol red-free RPMI supplemented with 10% charcoal-stripped FBS. Shmac5 cells were maintained in Stemline Keratinocyte Medium II with Stemline Keratinocyte Growth Supplement and 2% FBS, BOB cells were maintained in Keratinocyte-SFM medium and 10% FBS. SKBR3, LIM1215, and A549 cells were maintained in RPMI medium supplemented with 10% FBS. All media reagents were purchased from Gibco (Thermo Fisher Scientific). CWR22 cells were received with thanks from Professor TG Pretlow (Case Western Reserve University Cleveland, OH). LIM1215 cells were received with thanks from Professor Sabine Tejpar (KU Leuven, Leuven, Belgium). LNCaP, Du145, PC3, 22RV1, SKBR3, and A549 were obtained from the ATCC, Shmac5 and BOB cells were obtained from Culture Collections UK Health Security Agency. Cells from passages 2 to 4 were short tandem repeat–authenticated and tested *Mycoplasma* negative (MycoAlert, LONZA) at the beginning of the project. Exponentially growing cells were plated to achieve consistent confluency (80%–90%) and siRNAs (Thermo Fisher Scientific/Ambion Silencer, siRNA IDs: CTL—AM4611, *GRM1*—4392420; Horizon Discovery/Dharmacon OnTarget Plus (PerkinElmer), siRNA IDs: *HER2*—L-003126-00-0005, *HER3*—M-003127-03-0005) were then transfected the next day (final concentration: 10 nmol/L) using Lipofectamine RNAiMax (Invitrogen, Thermo Fisher Scientific) according to the manufacturer's instructions. DS-7423 (Daiichi Sankyo), lapatinib (Selleck Chemicals), VCC185369 (Vichem Chemie Ltd.), CPCCOEt (SML1124, Merck), GDC-0068 and GDC-0941 (Genentech), and Everolimus (StemCell Technologies) were kept in the medium for the duration of the culture experiment. MG132 proteasome-inhibitor (Merck) was added to the media 4 hours prior to the collection of cell lysates.

### Plasmids and generation of stable cell lines

LNCaP-HER3KD cell line was generated by lentiviral transduction using the pGIPZ system (Dharmacon) in combination with the second-generation packaging vectors pMDG.2 (for VSVG) and pCMVΔ8.91R (for gag pol). PEI-transfected HEK293T cells were used as packaging system and stable cultures of virally transduced LNCaP cells were established by selection in RPMI-10% FBS supplemented with 1 μg/mL puromycin.

### qRT-PCR

RNA was extracted using the RNeasy Mini Kit (Qiagen) according to manufacturer's protocol. RNA concentration and integrity were measured using a NanoDrop 1000 spectrophotometer (Thermo Fisher Scientific). A total of 500 ng RNA was transcribed using SuperScript III Reverse Transcriptase (Invitrogen, Thermo Fisher Scientific), according to manufacturer's instructions. The resulting cDNA was used as template for qPCR and amplified with the *Power* SYBR Green PCR Master Mix (Life Technologies, Thermo Fisher Scientific). Three independent experiments each with triplicate reactions were performed and analyzed using a 7500 Real-Time PCR System (Applied Biosystems). Relative quantification of the expression levels was determined using the ΔΔ*C*_t_ method and normalized to GAPDH transcript amplification.

### Immunoblotting

Cells were washed with ice-cold PBS, harvested by scraping and then homogenized in CelLytic M lysis buffer (Sigma, Merck) supplemented with phosphatase and protease inhibitors (PhosSTOP and cOmplete, Roche). Lysates were incubated (1 hour, 4°C) and then centrifuged at 14,000 × *g* (10 minutes, 4°C). Protein samples were quantified using the Pierce BCA Protein Assay (Thermo Fisher Scientific), resolved by SDS-PAGE and transferred to polyvinylidene difluoride membranes (Millipore, Merck). Membranes were blocked with Tris-buffered saline/0.5% Tween 20 (TBST, pH 7.5) containing 5% skim milk powder and probed overnight with primary antibodies against the following targets: PSMA (1H8H5, Thermo Fisher Scientific), EGFR (D38B1), HER2 (44E7), HER3 (D22C5), p-AKT(S473) (587F11), p-AKT (T308) (244F9), AKT (D38B1), p-S6 (S235/236) (D57.2.2E), Calnexin (C5C9) and Tubulin (9F3) were from Cell Signaling Technology, AR (Santa Cruz Biotechnology), GAPDH (GT239, GeneTex), and mGluR1 (07-617, Merck). Following incubation with horseradish peroxidase–linked secondary antibodies (Dako, Agilent Technologies), immunoreactive bands were visualized with Pierce ECL Western Blotting Substrate (Thermo Fisher Scientific).

### Immunoprecipitation

Cells were harvested, pelleted by centrifugation at 1,000 × *g* for 3 minutes, and washed with ice-cold PBS before lysis in immunoprecipitation buffer (IP buffer: 20 mmol/L Tris, 150 mmol/L NaCl, 10% glycerol, 1% NP-40, 5 mmol/L EDTA, 0.5 mmol/L EGTA, supplemented with phosphatase and protease inhibitors at pH 8.0). Lysates were centrifuged for 10 minutes at 16,000 × *g* at 4°C and precleared for 2 hours with protein G-Agarose (Merck), before overnight incubation with HER2 antibody. Protein G-agarose was then added to the lysates before an additional incubation for 5 hours. Beads were washed three times with IP buffer and once with IP buffer containing 1 mol/L NaCl, and the samples were then analyzed by SDS-PAGE as describe above.

### Clonogenic survival assays

Cells were seeded in p6 plates, allowed to adhere for 24 hours and then treated with drugs for 48 hours, as indicated above. Cells were subsequently trypsinized and seeded in 6-well plates at predetermined densities (400 CWR22 cells/well and 500 22RV1 cells/well) in drug-free medium for 2 weeks. Then, cells were fixed with MeOH/acetic acid (3:1) solution for 5 minutes, followed by staining with 0.5% crystal violet (Sigma, Merck) for 15 minutes. The number of colonies was determined by counting, and the surviving fraction was calculated using the plating efficiencies of the corresponding nontreated controls as a reference.

### Immunofluorescence

Cells were fixed in 4% PFA for 20 minutes at room temperature, permeabilized with 0.5% Triton X-100 and then blocked with 1% BSA/1% goat serum/PBS for 1 hour at room temperature. Sections were then incubated with HER2, HER3, and AR-targeting primary antibodies in blocking buffer overnight at 4°C in a humidified chamber. Subsequently, the samples were incubated with secondary antibodies with the appropriate fluorophore (Alexa Fluor, Thermo Fisher Scientific) were incubated for 2 hours at room temperature. Hoechst 33342 (Life Technologies, Thermo Fisher Scientific) was applied for 30 minutes at room temperature. Images were acquired using a Zeiss LSM880 with Airyscan confocal microscope (Zeiss).

### FRET analysis

Processing of cells for FRET determination by FLIM to quantify HER heterodimers was performed as previously described using an “open” automated FLIM microscope (24).

### Statistical analysis

Graphs and statistical analyses for all figures were performed using GraphPad Prism software. All results represent the mean ± SEM, unless stated otherwise. The significance of differences between the means or the population distributions was determined using two-way ANOVA with Dunnett test *post hoc* correction (xenograft tumor growth and clonogenic survival assays), two-way ANOVA with Tukey test *post hoc* correction (FRET assay) or two-tailed unpaired Student *t* test (for Western blotting and qRT-PCR assays). For all tests, differences were considered statistically significant if *P* values were < 0.05. *P* <0.05, <0.01, and <0.001 were indicated with *, **, and ***, respectively, in the figures.

### Data availability

The data generated in this study are available upon request from the corresponding author.

## Results

### Dual PI3K–mTORC1/2 inhibition differentially impacts on HER2/HER3 expression in prostate cancer cells according to PTEN status

**Figure 1.**
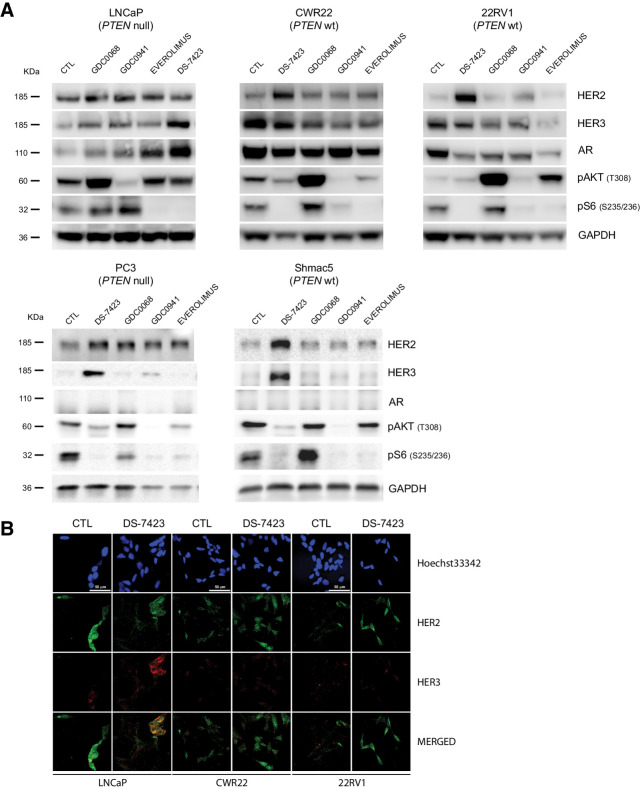
Expression profile for indicated proteins in prostate cancer cells after treatment with PI3K, mTOR, or dual inhibitors for 48 hours. **A,** DS-7423 increased expression of HER2 (4.5-fold in CWR22, 7.3-fold in 22RV1, 1.8-fold in PC3, and 1.2-fold in Shmac5 cells), HER3 (5-fold in LNCap, 1.3-fold in CWR22, 18-fold in PC3, and 4.5-fold in Shmac5 cells), and AR (6.1-fold in LNCaP). **B,** Immunofluorescence images show the DS-7423–induced upregulation of HER2 (green) and HER3 (red) in CWR22 and 22RV1 cells. Nucleus stained with Hoechst33342 (blue); scale bar, 50 μm.

On the basis of previous reports describing increased HER3 expression in response to PI3K inhibition in the PTEN-null cell line LNCaP (25), we aimed to expand the analysis to HER2, having previously shown that HER2-HER3 dimerization is crucial for cancer progression and patient stratification (24). We used the compound DS-7423 (Daiichi Sankyo), a dual inhibitor for PI3K and mTORC1/2, which blocks feedback loops leading to hyperactivation of different branches of the pathway (26). In addition, we also used the established inhibitors GDC-0941 (pan-class I PI3K), GDC-0068 (AKT), and Everolimus (mTORC1). As expected, DS-7423-treatment of LNCaP, PC3 and Shmac5 cells inhibited activation of AKT and increased expression of HER3 (Fig. 1; Supplementary Fig. S1). LNCaP cells also upregulated AR (Fig. 1A; Supplementary Fig. S2A). The link between HER3 and AR in LNCaP cells was further confirmed in HER3-depleted cells (Supplementary Fig. S2B), where increased expression of AR upon PI3K inhibition was abolished (Supplementary Fig. S2C), suggesting that HER3 would be upstream of transcriptional regulation of AR. Interestingly, cell lines CWR22 (androgen-dependent), its derived 22RV1 (androgen-independent) or PC3 (PTEN null) and Shmac5 (PTEN WT) exhibited substantial increase in expression of HER2 upon DS-7423 treatment (Fig. 1A; Supplementary Fig. S2D and S2E). In addition, dimer FRET analysis confirmed that blockade of the PI3K/AKT/mTOR pathway by DS-7423 alters HER2 dimer formation with other members of the HER family (EGFR and HER3), particularly after combined treatment of DS-7423 with lapatinib (EGFR/HER2 inhibitor; Supplementary Fig. S2F–S2G), thus highlighting the importance of receptor dimerization in HER signaling. Overall, our data show that the dual PI3K-mTORC1/2 inhibitor DS-7423 has the capacity to differentially impact HER2-associated signaling in prostate cancer cell lines according to their PTEN status.

### Dual PI3K–mTORC1/2 inhibition differentially impacts on PSMA expression in prostate cancer cells according to PTEN status

**Figure 2.**
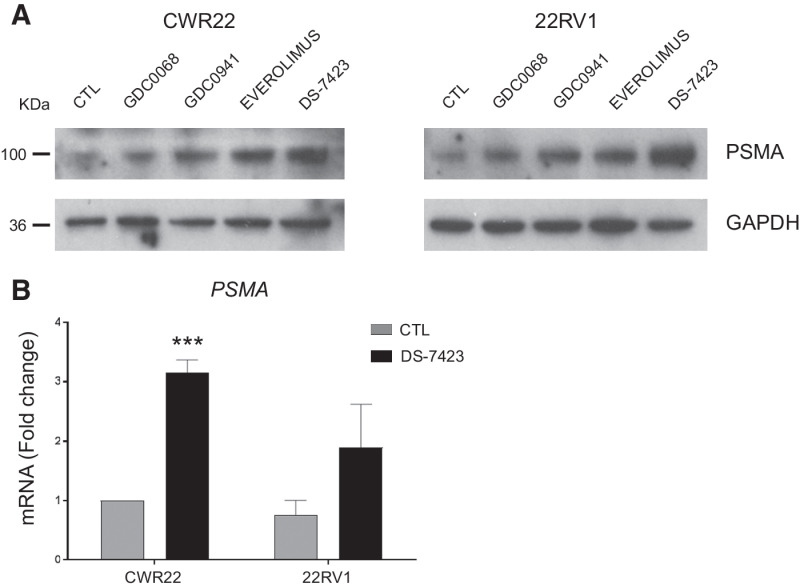
**A,** Modulation of expression of PSMA protein in CWR22 and 22RV1 cells by inhibitors with maximum upregulation induced by DS-7423 (3.4- and 5.6-fold, respectively). **B,** Detection of *PSMA* mRNA after treatment with DS-7423. Results are shown as mean with SE (*n* = 3; ***, *P* < 0.001).

Recent studies have revealed that the prognostic prostate cancer biomarker PSMA (through its interaction with scaffolding protein RACK1) can activate the PI3K-AKT pathway (27). We therefore aimed to evaluate PSMA expression upon PI3K/mTOR inhibition as a possible feedback mechanism. Similarly to HER2, PSMA expression was markedly increased in CWR22 and 22RV1 PTEN-wt cell lines (Fig. 2A). Gene expression analysis confirmed elevated *PSMA* mRNA levels (Fig. 2B) upon DS-7423 treatment. Furthermore, in a CWR22 xenograft model, DS-7423 treatment resulted in enhanced binding of the Gallium-68-PSMA tracer to the tumor (Supplementary Fig. S3). Taken together, these results showed enhanced PSMA expression upon PI3K-AKT-mTOR blockade and its positive association with HER2 levels in PTEN-wt prostate cancer cell lines.

### PSMA increase upon DS-7423 treatment is partially dependent on HER2 signaling

**Figure 3.**
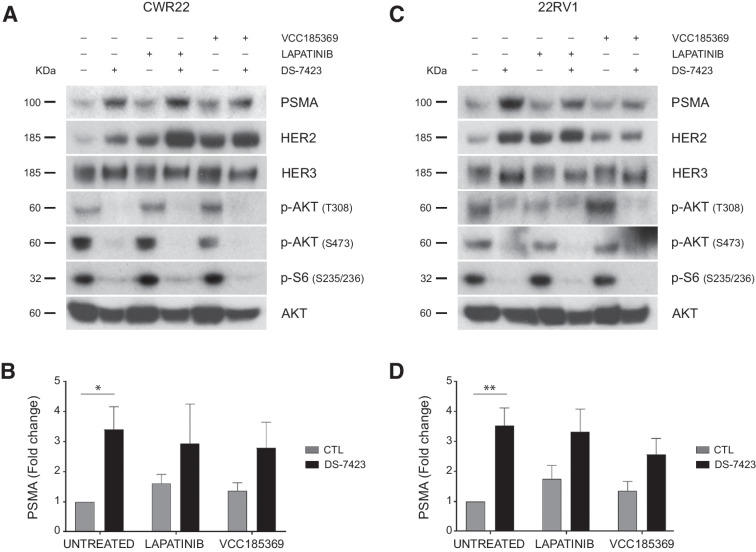
**A** and **C,** Expression profile of indicated proteins in CWR22 and 22RV1 cells after treatment with inhibitor alone or in combination. **B** and **D,** Quantification of PSMA expression in cells from **A** and **C**. Results are shown as mean with SE (*n* = 3; *, *P* < 0.05; **, *P* < 0.01).

Having demonstrated a correlation between HER2 and PSMA levels upon PI3K/mTOR inhibition, we questioned whether PSMA expression was dependent on HER2 signaling in PTEN-wt prostate cancer cells. DS-7423-dependent changes in PSMA levels were partially abrogated by treatment with lapatinib or VCC185369 (28), an additional inhibitor of HER2 kinase activity (Fig. 3), demonstrating the involvement of HER2 signaling in PSMA upregulation upon PI3K/mTOR inhibition in PTEN-wt prostate cancer cell lines.

### PSMA activity enhances HER2 expression through mGluR1 signaling in a feedback loop mechanism

**Figure 4.**
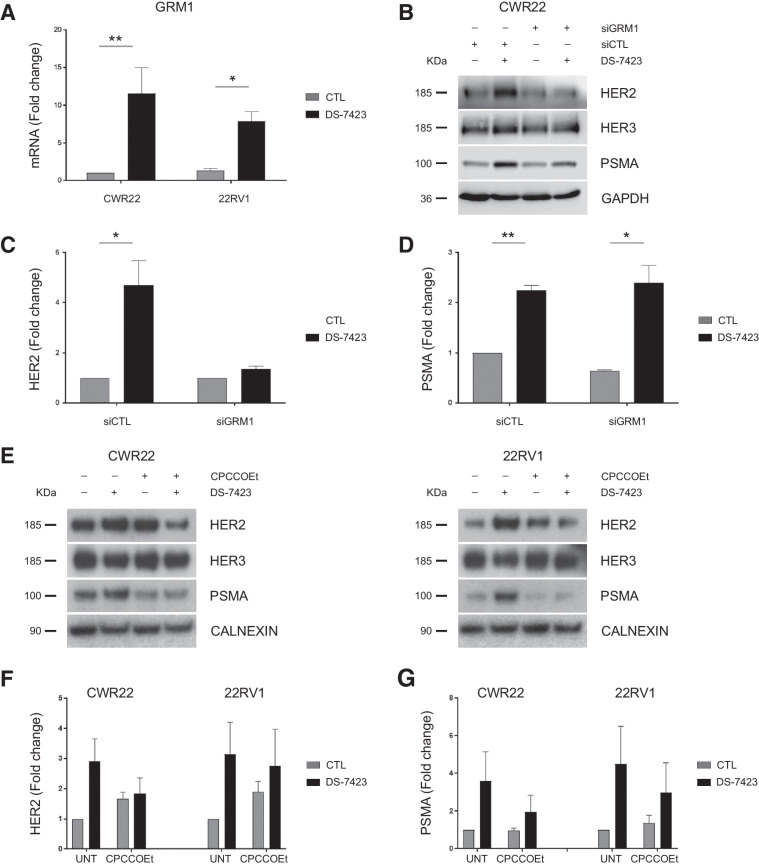
**A,** qRT-PCR analysis of GRM1 gene expression in CWR22 and 22RV1 cells. Results are shown as mean with SE (*n* = 3; *, *P* < 0.05; **, *P* < 0.01). **B,** Expression of HER2, HER3, and PSMA in control and GRM1-depleted CWR22 cells. **C** and **D,** Quantification of HER2 and PSMA expression in cells from **B**. Results are shown as mean with SE (*n* = 3; *, *P* < 0.05; **, *P* < 0.01). **E,** Expression of of HER2, HER3, and PSMA after treatment with inhibitor alone or in combination. **F** and **G,** Quantification of expression of HER2 and PSMA presented in **E**. Results are shown as mean with SE (*n* = 3).

**Figure 5.**
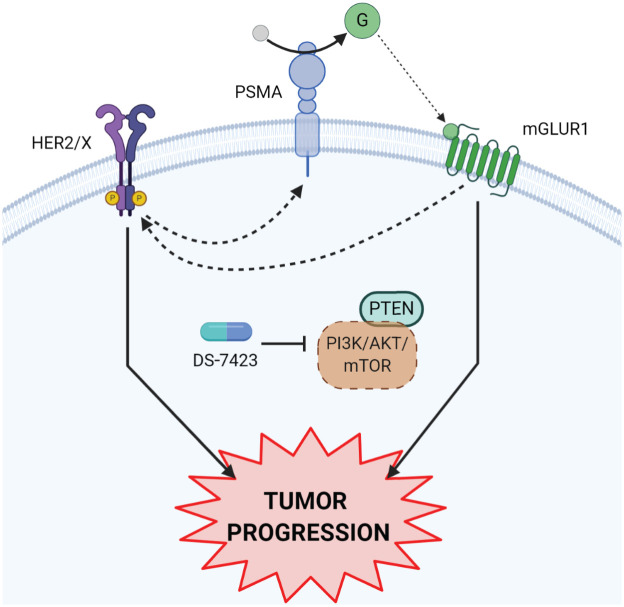
Model proposed describing the PSMA-mGluR1-HER2 mechanism of resistance to dual PI3K-mTOR inhibitor DS-7423 (created with BioRender.com). HER2/X represents the HER2-containing receptor protein complex (X = EGFR or HER3 in Supplementary Fig. S1E and S1F). A feedback mechanism that is activated in response to PI3K/mTOR inhibition involves upregulation of ErbB/HER protein (HER2 or HER3) according to the genetically diverse prostate cancer cell lines we have tested. Downstream effects include a PSMA increase upon DS-7423 treatment that is partially dependent on HER2 signaling. Targeting the PI3K/mTOR pathway has unraveled a complex relationship between PSMA increase and HER2 upregulation via mGluR1 activity (Fig. 4B).

PSMA enzymatic activity triggers the release of glutamate and the activation of protumorigenic signaling pathways (including ERK, PKC, and PI3K) that depend on the binding of glutamate to the metabotropic glutamate receptor mGluR1 (7, 8). Therefore, we speculated that increased HER2 levels were a consequence of enhanced mGluR1-driven signaling. *GRM1* mRNA and protein levels were increased upon DS-7423 treatment (Fig. 4A; Supplementary Fig. S4A) in both CWR22 and 22RV1 PTEN-wt cell lines. mGluR1 depletion (Supplementary Fig. S4B) impaired HER2 upregulation upon PI3K/mTOR inhibition (Fig. 4B and C) in a similar fashion as observed upon HER3 knockdown (Supplementary Fig. S4C and S4D) without affecting PSMA expression (Fig. 4B and D). Mechanistically, both HER3 and mGluR1-depleted cells displayed enhanced HER2 ubiquitylation suggesting that mGluR1 (and HER3) are important for HER2 protein stability (Supplementary Fig. S4E). Moreover, the mGluR1 non-competitive antagonist CPCCOEt displayed similar effects by diminishing DS-7423–induced changes in HER2 expression (Fig. 4E and F). Pharmacologic inhibition of mGluR1 also abrogated differences in PSMA expression upon PI3K/mTOR blockade (Fig. 4E and G). Collectively, our data suggest that PTEN-wt cell lines respond to DS-7423 treatment by upregulating mGluR1 and HER2, whose pathways (through regulation of PSMA expression) enhance each other through a positive feedback mechanism that promotes acquisition of resistance to PI3K/mTOR inhibition (Fig. 5).

### Combination of HER/mGluR1 inhibition with DS-7423 treatment reduces the tumorigenic potential of PTEN-wt prostate cancer cell lines

**Figure 6.**
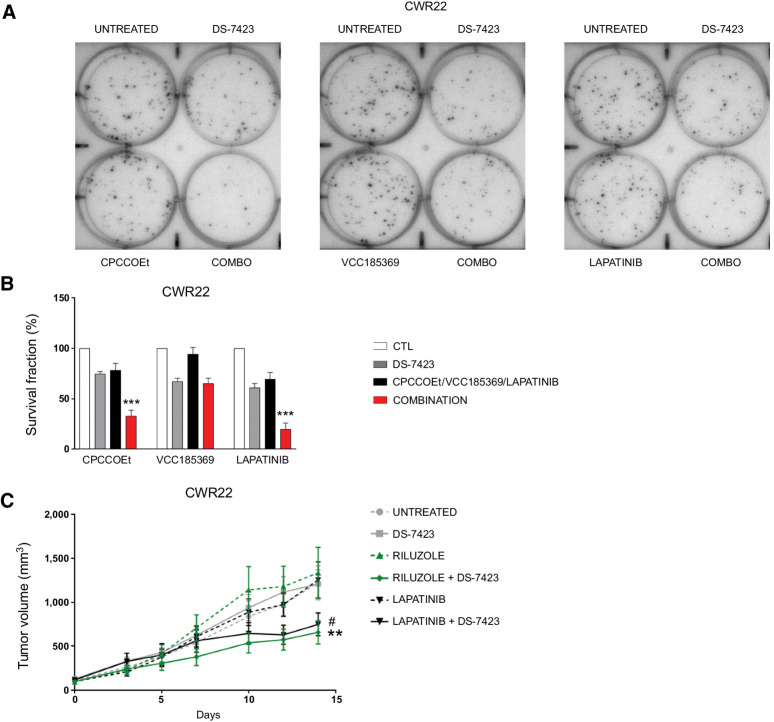
**A,** Representative images of clonogenic survival assays in CWR22 cells treated with CPCCOEt, VCC185369, and lapatinib alone or in combination with DS-7423 inhibitor. **B,** Quantification of clonogenic survival assays from **A**. Results are shown as mean with SE (*n* = 3; ***, *P* < 0.001). **C,** Tumor volume growth curves of CWR22 xenografts for the indicated treatments. Results are shown as mean with SE (*n* = 4–5; #, *P* = 0.06; **, *P* < 0.01). For those individuals that were culled before the end of the experiment due to ethical limits, a growth constant was calculated as (log fold change), and the tumor volume was estimated to obtain the average.

Given that our data suggest that PTEN-wt prostate cancer cell lines rely on mGluR1 and HER2 to overcome PI3K/mTOR inhibition, we sought to evaluate whether simultaneous inhibition of both signaling pathways would impact on the tumorigenic potential of these cell lines. Combination treatment with DS-7423 and either CPCCOEt or lapatinib resulted in reduced clonogenic survival in both PTEN-wt androgen-dependent (CWR22) and androgen-independent (22RV1) cells when compared with single-agent treatments (Fig. 6A and B; Supplementary Fig. S5A and S5B). Furthermore, CWR22 xenografts treated with a combination of PI3K/mTOR inhibitor and either Riluzole (blocking agent for glutamate release) or lapatinib showed significant reduction in tumor volume after a 2-week treatment, when compared with their single-agent controls (Fig. 6C; Supplementary Fig. S5C). We also found that combination of DS-7423 and CPCCOEt (Supplementary Fig. S6) or with lapatinib (Supplementary Fig. S7) also led to enhanced cytotoxic effect in other prostate and other types of cancer cells. Overall, the combination of *in vitro* and *in vivo* data confirms that blockade of the PI3K/mTOR pathway in combination with mGluR1 or HER inhibition negatively affects tumor progression. While analysis of The Cancer Genome Atlas dataset has revealed there is an interesting interaction between PTEN status with GRM1 in dictating prognostic outcomes (Supplementary Fig. S8), further work including clinical trials will be required to link this prognostic effect to a possible stratification strategy to achieve better clinical outcome when PI3K/mTOR inhibition is applied to treat patients with metastatic prostate cancer.

## Discussion

Innovations in treatment, imaging, and molecular characterization of advanced and metastatic prostate cancer have considerably improved outcomes of patients in recent years. However, despite initial good responses to AR-targeting agents and chemotherapy, the majority of metastatic prostate cancer ultimately develops into castration resistant disease. Several novel potent antiandrogen agents and chemotherapy have proven benefit for such disease and are now part of standard of care in this setting (29–31). In addition, PARP inhibitors (FDA-approved), immunotherapy-based approaches (CAR-T, BiTE, PD-1 inhibition), PSMA-based radioligand targeted therapy and PI3K-AKT-mTOR targeted therapy are all under clinical evaluation (32).

In the complex genomic landscape of prostate cancer, the PI3K-AKT-mTOR pathway is one of the most frequently activated signaling pathways and PTEN one of the most consistently altered genes. Indeed, PTEN loss is associated with events occurring early in prostate cancer development, and is strongly linked to advanced prostate cancer progression and poor clinical outcome (33). In addition to its role in the PI3K pathway, PTEN plays an important role in the regulation of the tumor microenvironment cross-talk, DNA damage, immune response, and inflammation (34). Moreover, prolonged inhibition of AR triggers a compensatory activation of the PI3K pathway, most often due to the genomic loss of PTEN, driving progression to CRPC (35). However, there are scenarios where hyperactivation of PI3K-AKT-mTOR signaling, resulting from alternative mechanisms, is observed in a normal PTEN background (21). Consequently, the PTEN status should be considered when analyzing the efficacy of the PI3K-AKT-mTOR targeted therapies in the clinical setting (15, 36).

Resistance mechanisms to PI3K-AKT-mTOR inhibition and strategies to overcome these have already been documented in PTEN-mutant prostate cancer (25, 37). However, it remains unclear whether PI3K-AKT-mTOR inhibition would be of benefit in a PTEN-wt background. The results of our work demonstrate a differential HER receptor response to the dual PI3K-mTOR inhibitor DS-7423 depending on the PTEN status of the prostate cancer cells. PTEN-wt PCa CWR22 and its CRPC 22Rv1 derivative upregulated HER2 while PTEN-null PC cells upregulated HER3. The association between HER2 and PI3K pathways has been described extensively in breast cancer (38). However, irrespective of the specific HER family member being upregulated, our expertise suggests that it is the dimerization dynamics between receptor partners (EGFR, HER2, and HER3, Supplementary Fig. S2E and S2F) that is crucial in both PTEN-wt and -null genomic contexts (39). Interestingly, PTEN-wt prostate cancer cells also displayed increased levels of PSMA upon DS-7324 treatment, correlating with increased HER2 expression, as documented in the context of HER2^+^ breast cancer (40). The observed PSMA increase upon PI3K/mTOR inhibition was partially abrogated by the HER2 inhibitors lapatinib and VCC185369, a finding that constitutes the first evidence of HER-dependent PSMA regulation, an avenue that warrants further exploration. PSMA has, however, been shown to allow the activation of EGFR through the assembly of a multi-protein signaling complex in prostate cancer cell lines (41). PSMA has been extensively studied for (i) its capacity to drive endocytosis and thereby provide the basis for PSMA-bound therapeutics, including chemoradiotherapy and HER-targeted therapies (42) and (ii) its enzymatic activity, releasing glutamate that activates mGluR GPCR-dependent signaling (8), whose role in cancer has been described extensively (9). mGluR1 signaling-associated pathways include PI3K-AKT-mTOR itself but also ERK1/2 (43–45) as potential pathways to overcome PI3K inhibition. In addition to PSMA expression, mGluR1 levels were elevated upon PI3K-mTOR inhibition. Silencing of mGluR1 (via genetic or pharmacologic approaches) resulted in a decrease in HER2 and PSMA expression levels. Cross-talk between metabotropic glutamate receptors and HER family receptors has already been demonstrated for mGluR5 and EGFR in the central nervous system (46). mGluR1 expression levels and activity are tightly controlled by neuregulin-dependent HER receptor signaling in rodent neurons (47). However, no evidence of mGluR1-dependent HER receptor activity has been described thus far. While direct modulation of phosphorylation status of mGluR1 by tyrosine kinase(s) (ErbB receptors; ref. 48) and modulation of their function was suggested previously, there is also a possibility that activation of mGluR1 may lead to the activation of the ERK1/2 pathway (49) which lies downstream of ErbB2. Finally, mGluR1-related Ca^2+^ signaling controls PSMA transcription (6).

In conclusion, we have described a novel resistance mechanism to PI3K-AKT-mTOR inhibition in PTEN-wt prostate cancer cell lines, involving HER receptors, PSMA and mGluR1 in a regulatory loop (Fig. 5). Although further research is needed to elucidate the regulatory dynamics among the different components of this network, targeting PI3K and HER2/mGluR1 pathways represents an intriguing therapeutic possibility, as also suggested by previous evidence (45). Indeed, PTEN-wt CWR22 xenografts displayed reduced *in vivo* growth following treatment with a combination of DS-7423 and lapatinib or DS-7423 and Riluzole, when compared single-agent treatments. This work therefore suggests a novel combination strategy for PI3K inhibitors to benefit patients with normal PTEN activity.

In addition to the above combination strategies, the use of PSMA-based imaging and radio-ligand therapy is in the forefront of evaluation and interest in personalized treatment in prostate cancer. Our observations regarding the differential expression of PSMA upon PI3K inhibition based on PTEN status, and especially the increase in PSMA expression in PTEN-wt prostate cancer cells treated with DS-7423 suggest that this glycoprotein can be utilized as a predictive biomarker of resistance to treatment.

## Authors' Disclosures

P.R. Barber reports grants from Cancer Research UK during the conduct of the study; personal fees from Oxford Optronix Ltd and other support from Nano Clinical Ltd outside the submitted work. K. Ng reports personal fees from Pfizer, GSK/Tesaro, and Boehringer Ingleheim outside the submitted work. B. Vanhaesebroeck reports personal fees from Pharming Leiden (the Netherlands), Olema Pharmaceuticals, iOnctura, and Venthera outside the submitted work. S. Chowdhury reports personal fees from AAA, Janssen, Bayer, Astellas, Athenex, AZ, TELIX, Remedy, and Huma outside the submitted work. T. Ng reports other support from Nanoclinical Ltd (CMO) outside the submitted work. No disclosures were reported by the other authors.

## Supplementary Material

Supplementary Figure

Supplementary Figure

Supplementary Figure

Supplementary Figure

Supplementary Figure

Supplementary Figure

Supplementary Figure

Supplementary Figure
