# Supplementary material for: HER2 Mediates PSMA/mGluR1-Driven Resistance to the DS-7423 Dual PI3K/mTOR Inhibitor in PTEN Wild-type Prostate Cancer Models
Source: Mol Cancer Ther. 2022 Jan 27;21(4):667–76. doi: 10.1158/1535-7163.MCT-21-0320 (PMC7612588; doi:10.1158/1535-7163.MCT-21-0320)
Supplement: Supplementary Figure [file mct-21-0320_supplementary_figure_2_supp2.pdf]

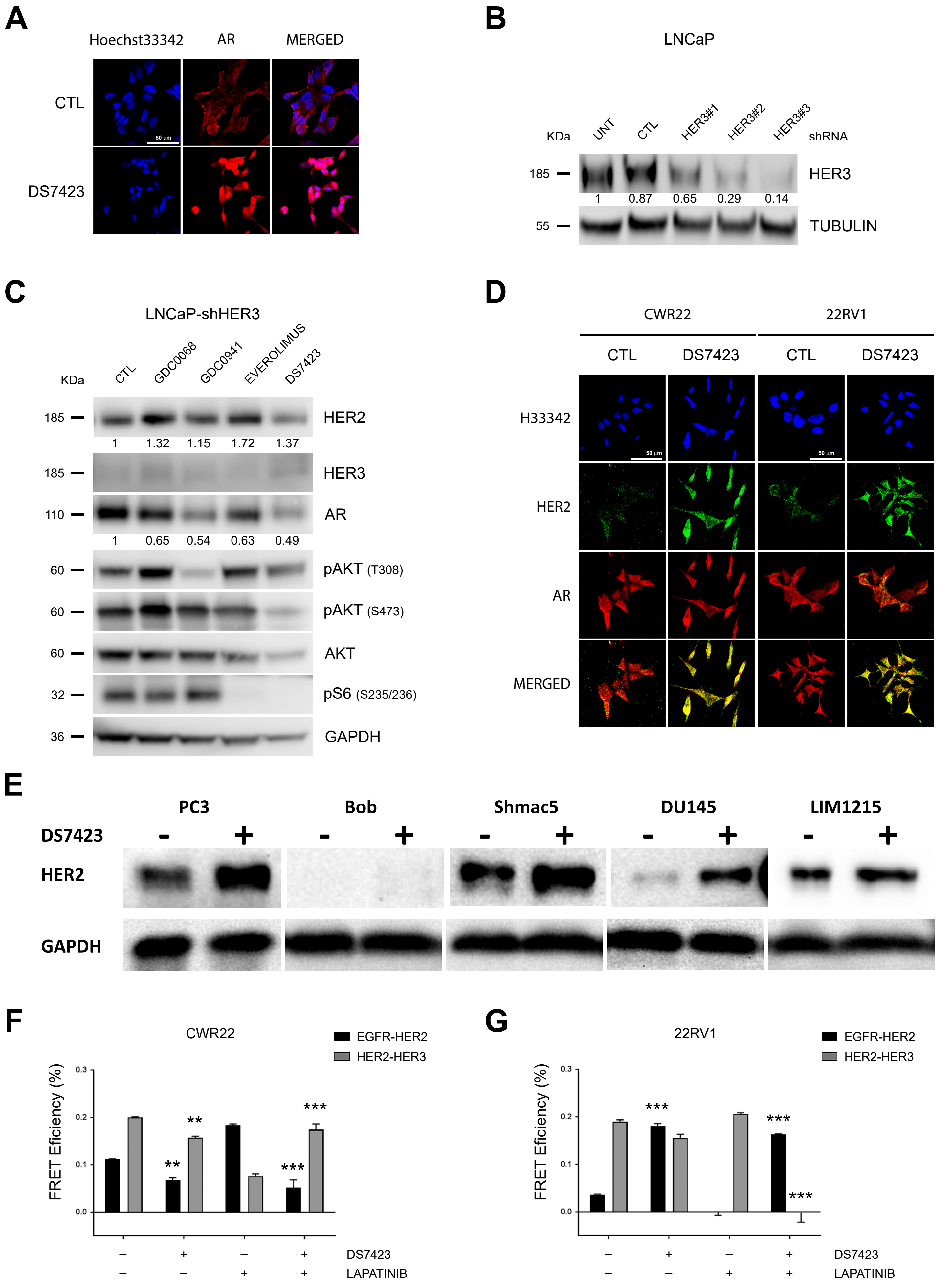
A
Hoechst33342
AR
MERGED
CTL
DS7423
50 μm
B
LNCaP
KDa
UNT
CTL
HER3#1
HER3#2
HER3#3
shRNA
185
HER3
1
0.87
0.65
0.29
0.14
55
TUBULIN
C
LNCaP-shHER3
KDa
CTL
GDC0068
GDC0941
EVEROLIMUS
DS7423
185
HER2
1
1.32
1.15
1.72
1.37
185
HER3
110
AR
1
0.65
0.54
0.63
0.49
60
pAKT (T308)
60
pAKT (S473)
60
AKT
32
pS6 (S235/236)
36
GAPDH
D
CWR22
22RV1
CTL
DS7423
CTL
DS7423
H33342
HER2
AR
MERGED
50 μm
50 μm
E
PC3
Bob
Shmac5
DU145
LIM1215
DS7423
- +
- +
- +
- +
- +
HER2
GAPDH
F
CWR22
EGFR-HER2
HER2-HER3
FRET Eficiency (%)
0.3
0.2
0.1
0.0
**
**
***
***
–
+
–
+
DS7423
–
–
+
+
LAPATINIB
G
22RV1
EGFR-HER2
HER2-HER3
FRET Eficiency (%)
0.3
0.2
0.1
0.0
***
***
***
–
+
–
+
DS7423
–
–
+
+
LAPATINIB

Figure S2

Supplementary Figure 2.

A) Representative images AR (red) in control and DS-7423-treated LNCaP cells, nucleus stained with Hoechst33342 (blue), scale bar = 50 μm. B) Effect of the indicated *HER3* siRNAs on expression of HER3 in LNCaP cells. C) Expression profile in HER3- depleted LNCaP cells after treatment with indicated drugs. D) Representative images of HER2 (green) and AR (red) in control and DS-7423-treated CWR22 and 22RV1 cells. Nucleus stained with Hoechst33342 (blue), scale bar = 50 μm. E) HER2 upregulation in wide range of cells – PC3 90%, Shmac5 80%, DU145 600%, LIM1215 20%. FRET efficiency percentage for the dimers EGFR-HER2 (*black bars*) and HER2-HER3 (*grey bars*) in CWR22 (F) and 22RV1 (G) cells. Results are shown as mean with standard error (n=3, ** $p<0.01$, *** $p<0.001$).
