# Supplementary material for: HER2 Mediates PSMA/mGluR1-Driven Resistance to the DS-7423 Dual PI3K/mTOR Inhibitor in PTEN Wild-type Prostate Cancer Models
Source: Mol Cancer Ther. 2022 Jan 27;21(4):667–76. doi: 10.1158/1535-7163.MCT-21-0320 (PMC7612588; doi:10.1158/1535-7163.MCT-21-0320)
Supplement: Supplementary Figure [file mct-21-0320_supplementary_figure_3_supp3.pdf]

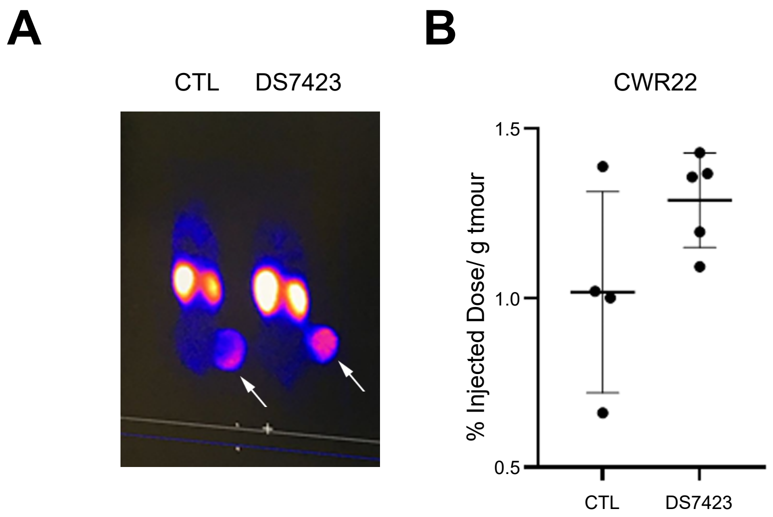

Supplementary Figure 3.
Representative PET imaging of radiotracer 68Ga-THP-PSMA uptake in CWR22 xenografts (white arrows) in untreated (left) or DS-7423 (right) mice (A) and quantification of PET imaging assays (B). Results are normalized to 1 untreated mouse are shown as mean with standard error along to individual values (n=4-5).
