# Supplementary material for: HER2 Mediates PSMA/mGluR1-Driven Resistance to the DS-7423 Dual PI3K/mTOR Inhibitor in PTEN Wild-type Prostate Cancer Models
Source: Mol Cancer Ther. 2022 Jan 27;21(4):667–76. doi: 10.1158/1535-7163.MCT-21-0320 (PMC7612588; doi:10.1158/1535-7163.MCT-21-0320)
Supplement: Supplementary Figure [file mct-21-0320_supplementary_figure_4_supp4.pdf]

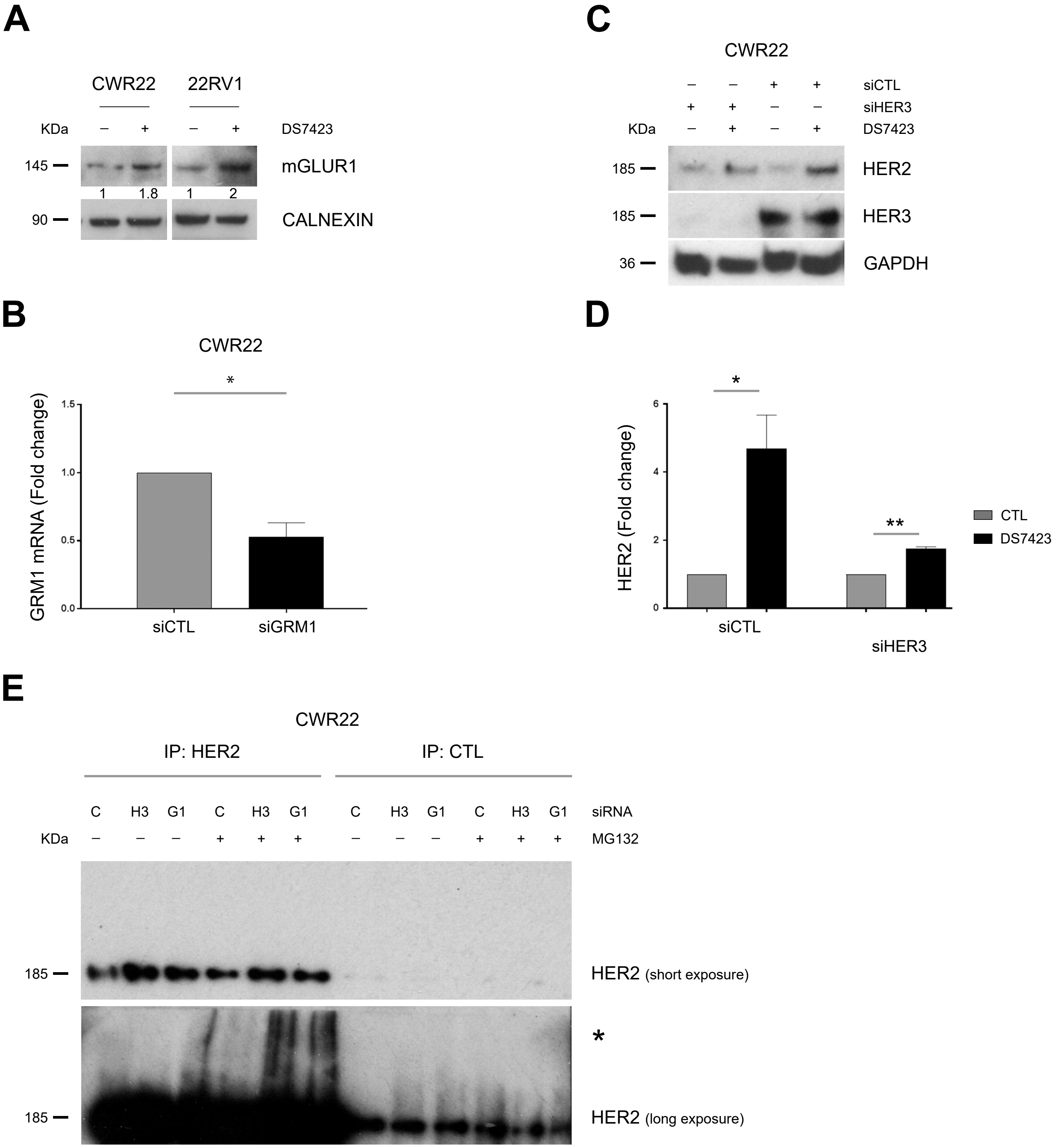

Supplementary Figure 4.
A) Expression of mGluR1 in CWR22 and 22RV1 cells treated with DS-7423. B) RT-qPCR analysis of GRM1 gene expression in control and siGRM1 transfected CWR22 cells. Results are normalized to the CWR22 siCTL culture and shown as mean with standard error (n=3; * $p<0.05$). C) Expression of HER2 in HER3-depleted CWR22 cells treated with DS-7423 and it's quantification (D). Results are normalized to the corresponding untreated CWR22 condition and shown as mean with standard error (n=3, * $p<0.05$, ** $p<0.01$). E) Expression of HER2 and ubiquitylated HER2 (star) in control (C), HER3-depleted (H3) and mGluR1-depleted (G1) in untreated and MG132-treated CWR22 cells following immunoprecipitation with anti-HER2 or control antibodies.
