# Supplementary material for: HER2 Mediates PSMA/mGluR1-Driven Resistance to the DS-7423 Dual PI3K/mTOR Inhibitor in PTEN Wild-type Prostate Cancer Models
Source: Mol Cancer Ther. 2022 Jan 27;21(4):667–76. doi: 10.1158/1535-7163.MCT-21-0320 (PMC7612588; doi:10.1158/1535-7163.MCT-21-0320)
Supplement: Supplementary Figure [file mct-21-0320_supplementary_figure_5_supp5.pdf]

A

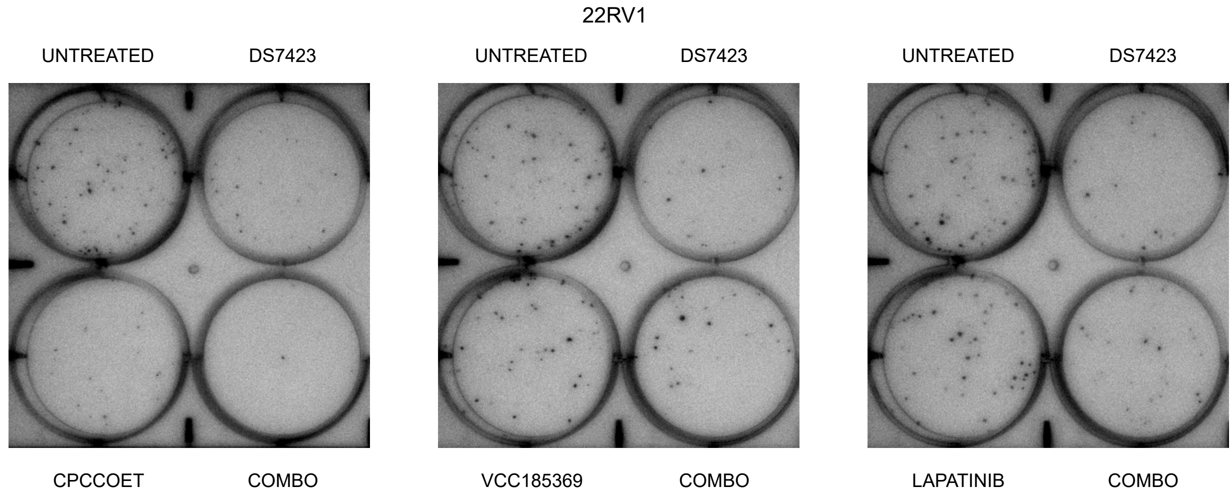


B

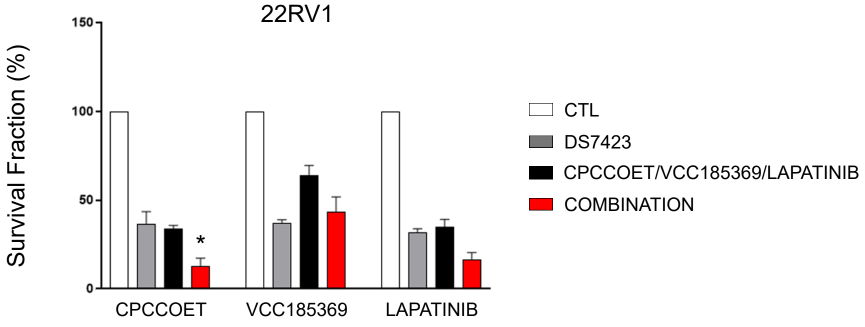


C

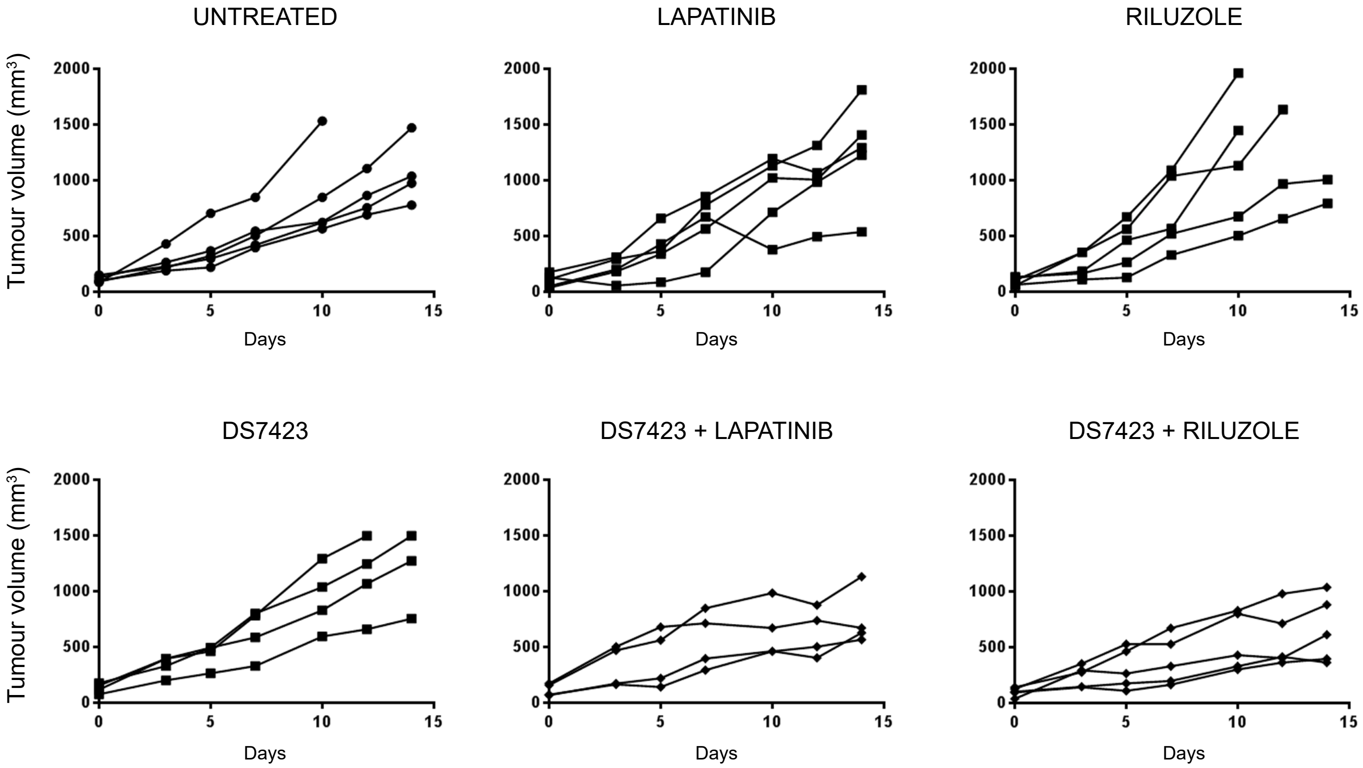


Figure S5

**Supplementary Figure 5.**

A) Representative pictures of clonogenic survival assays in 22RV1 cells treated with CPCCOET (*left panel*), VCC185369 (*middle panel*) and lapatinib (*right panel*) alone or in combination with DS-7423 PI3K/mTOR inhibitor. B) Quantification of clonogenic survival assays in A. Results are normalized to the untreated condition for each individual treatment and shown as mean with standard error (n=4-5, * $p<0.05$). C) Tumor volume growth curves of CWR22 xenografts for each individual mouse for the indicated treatments.
