# Supplementary material for: HER2 Mediates PSMA/mGluR1-Driven Resistance to the DS-7423 Dual PI3K/mTOR Inhibitor in PTEN Wild-type Prostate Cancer Models
Source: Mol Cancer Ther. 2022 Jan 27;21(4):667–76. doi: 10.1158/1535-7163.MCT-21-0320 (PMC7612588; doi:10.1158/1535-7163.MCT-21-0320)
Supplement: Supplementary Figure [file mct-21-0320_supplementary_figure_6_supp6.pdf]

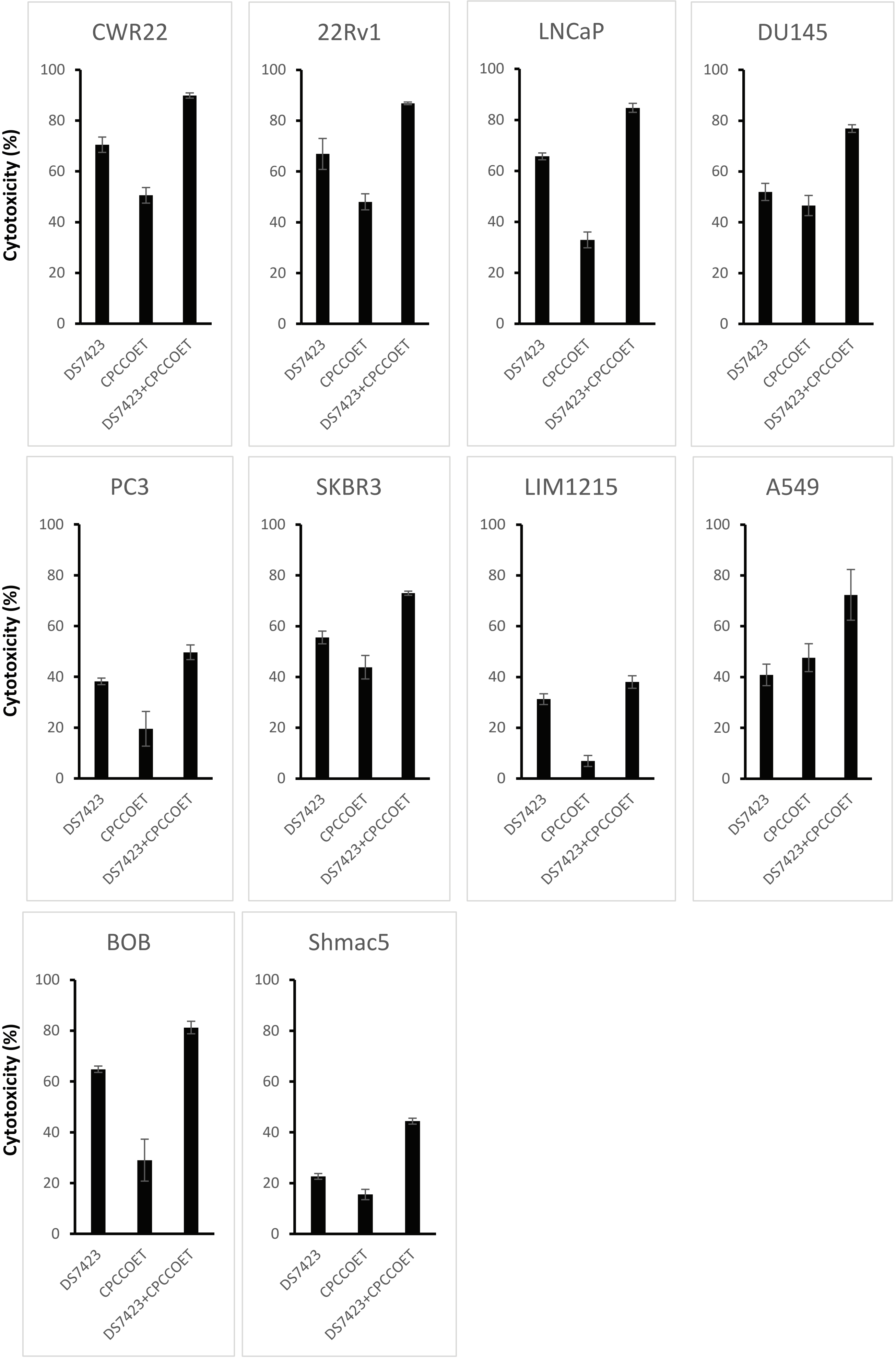
CWR22
22Rv1
LNCaP
DU145
PC3
SKBR3
LIM1215
A549
BOB
Shmac5
Cytotoxicity (%)
0
20
40
60
80
100
DS7423
CPCCOET
DS7423+CPCCOET

Figure S6

Supplementary Figure 6.

Cytotoxic effect measured with Alamar Blue of mGluR1 inhibition (CPCCOET) and PI3K/mTOR inhibition (DS7423), either alone or in combination, in different cell lines. Cytotoxicity response of CWR22, 22RV1 and LNCaP (prostate cancer cells, androgen positive), PC3, Shmac5, Bob and DU145 (prostate cancer cells, androgen negative), LIM1215 (colon cancer cells), A549 (lung cancer cells) and SKBR3 (breast cancer cells). The Y axis shows cytotoxicity (%) calculate according to the equation (1-AT/AC)*100, where AT is signal in treatment group and AC is a signal in control group. Data are expressed as Mean ± SD, the difference between DS7423 alone and in combination with CPCCOET is statistically significant for all cell lines ($P<0.0001$).
