# Supplementary material for: HER2 Mediates PSMA/mGluR1-Driven Resistance to the DS-7423 Dual PI3K/mTOR Inhibitor in PTEN Wild-type Prostate Cancer Models
Source: Mol Cancer Ther. 2022 Jan 27;21(4):667–76. doi: 10.1158/1535-7163.MCT-21-0320 (PMC7612588; doi:10.1158/1535-7163.MCT-21-0320)
Supplement: Supplementary Figure [file mct-21-0320_supplementary_figure_7_supp7.pdf]

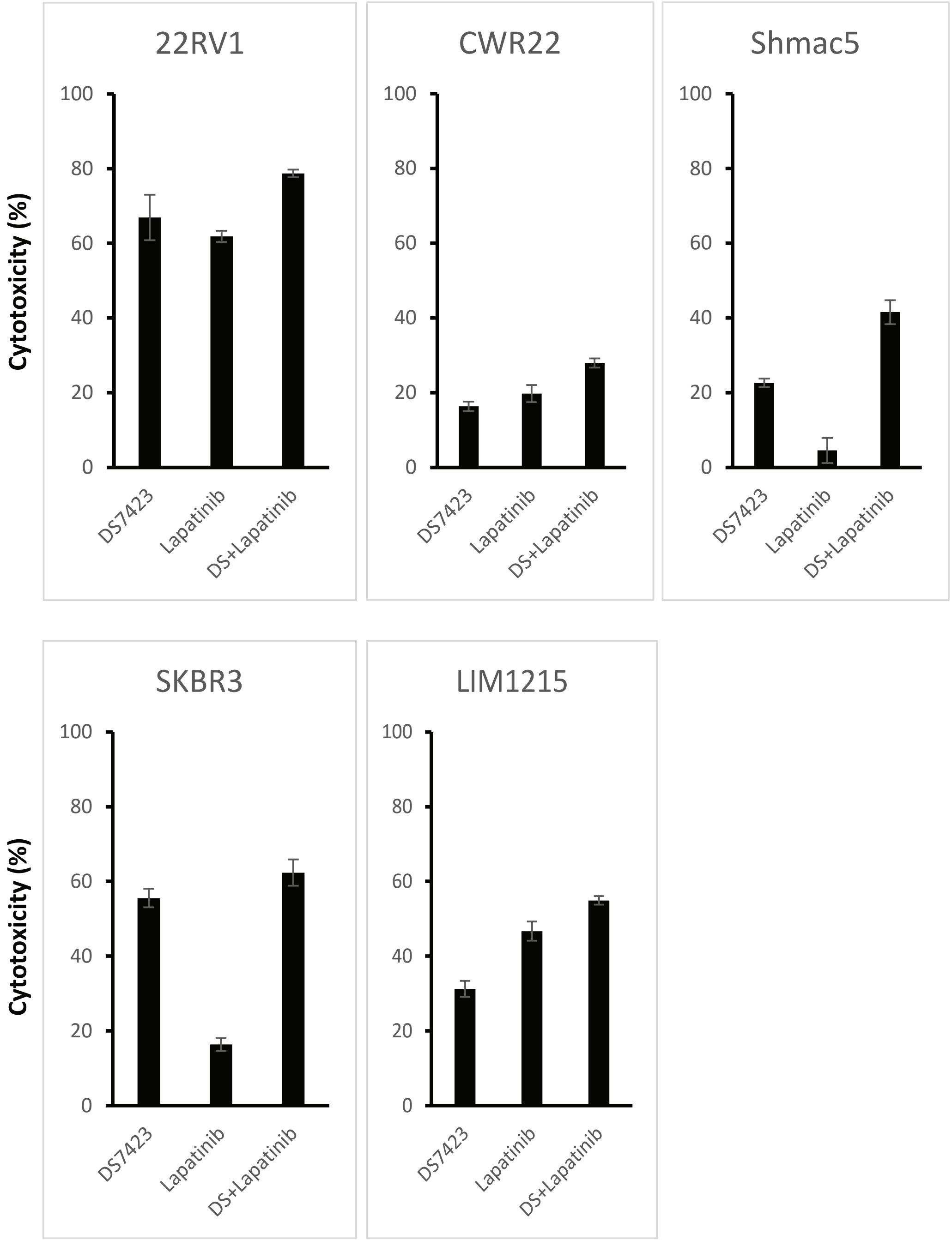

Supplementary Figure 7
Effect of DS7423 in combination with lapatinib in prostate, breast and colon cancer cells. Cytotoxicity response of CWR22, 22Rv1 and Shmac5 (prostate cancer cells), SKBR3 (breast cancer cells) and LIM1215 (colon cancer cells). Y axis show cytotoxicity % calculate according to the equation (1-AT/AC)*100, where AT is signal in treatment group and AC is a signal in control group. Data expressed as Mean ± SD, the difference between DS7423 and combination with lapatinib is statistically significant for all cell lines ($P<0.0001$).
