# Supplementary material for: HER2 Mediates PSMA/mGluR1-Driven Resistance to the DS-7423 Dual PI3K/mTOR Inhibitor in PTEN Wild-type Prostate Cancer Models
Source: Mol Cancer Ther. 2022 Jan 27;21(4):667–76. doi: 10.1158/1535-7163.MCT-21-0320 (PMC7612588; doi:10.1158/1535-7163.MCT-21-0320)
Supplement: Supplementary Figure [file mct-21-0320_supplementary_figure_8_supp8.pdf]

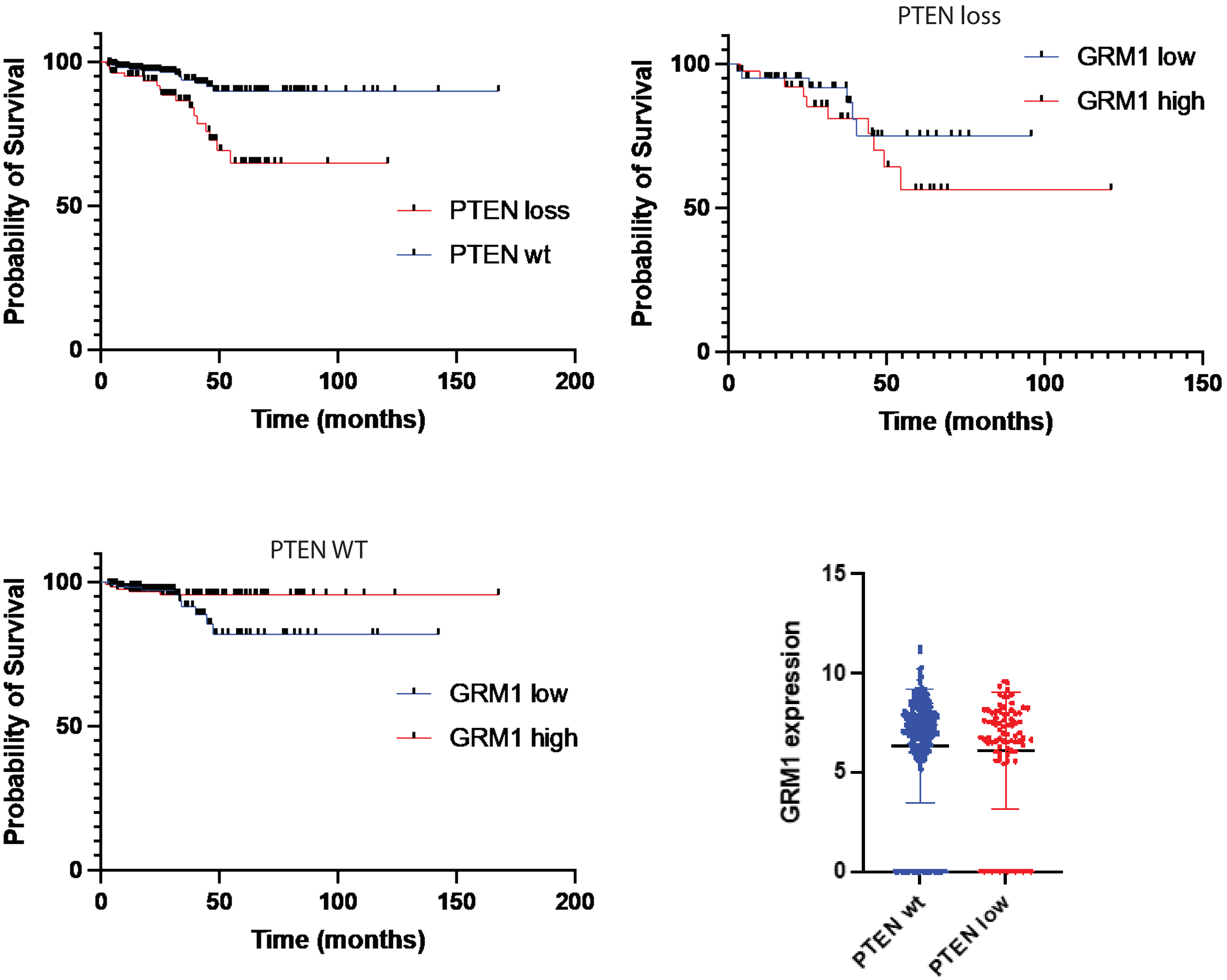

Supplementary Figure 8
A) Kaplian Meier plot showing overall survival of TCGA GDC PRAD patients who are PTEN wt (n=255) or carry a PTEN loss (n=82). Log Rank test; p=0.0005. B) Kaplian Meier plot showing overall survival of PTEN loss patients who express high (n=41) or low (n=41) GRM1. Log Rank test; p=0.3436.
C) Kaplian Meier plot showing overall survival of PTEN wt patients who express high (n=126) or low (n=127) GRM1. Log Rank test; p=0.182. D) Expression of GRM1 in PTEN WT and PTEN low in untreated patient populations
